# Psychosocial and auditory factors that influence successful music-based auditory training in pediatric cochlear implant recipients

**DOI:** 10.3389/fnhum.2023.1308712

**Published:** 2023-12-21

**Authors:** Kate Gfeller, Ruth Mallalieu

**Affiliations:** ^1^Department of Otolaryngology—Head and Neck Surgery, School of Music, Department of Communication Sciences and Disorders, The University of Iowa, Iowa City, IA, United States; ^2^Bodleian Libraries, The University of Oxford, Oxford, United Kingdom

**Keywords:** cochlear implants, pediatric, music perception, aural rehabilitation, music training

## Abstract

**Introduction:**

Cochlear implants (CIs), which are designed to support spoken communication of persons with severe to profound hearing loss, can provide improved hearing capability through passive exposure. However, auditory training may optimize perception of spectrally complex sounds such as music or speech. Reviews of music-based training for pediatric CI users have reported modest though variable benefits, as well as problems with attrition. It is presumed that more substantial changes may result from longer, more intensive training; however, the development of protocols sufficiently motivating for sustained intensity is challenging. This article examined the experiences of star pediatric CI users, whose years of music training have yielded exceptional auditory benefits. Greater understanding of their experiences and attitudes may suggest best practices for music-based training. Research aims included: (a) characterizing the musical behaviors and perceptual learning processes of music-centric (Music-centric, for purposes of this paper, refers to CI users who engage in sustained and successful music making such as music lessons and ensembles and focused music listening over a period of years, and who derive deep satisfaction from those experiences.) pediatric CI users, and (b) identifying psychosocial and auditory factors that motivated persistence in auditory training.

**Methods:**

We used qualitative and patient-engaged research methodologies, gathering data through questionnaires with open-ended questions. The participants, six music-centric CI users and five parents, described their experiences and attitudes regarding music training, and factors that supported or undermined those experiences. Data were analyzed using reflexive thematic analysis.

**Results:**

The codes were consolidated into five themes and organized into a Model of Music-Based Learning for Pediatric Cochlear Implant Recipients. Sustained participation in music training was perceived as a dynamic process including varied musical stimuli, and moderated by intrinsic (attitude, perceived behavioral control) and extrinsic (parents, teachers, peers) influences, hearing status, sound access and background factors.

**Discussion:**

These themes highlighted motivational factors that pediatric CI users and parents considered important to sustained, intensive and successful music learning throughout childhood and adolescence. These factors should be considered in the development of music-based training for pediatric CI recipients.

## 1 Introduction

*“For me, it started when I wanted to understand what music was like and so I turned on the family radio and tried to find a station with music. This must have been when I was 5 or 6. My older sibling asked me what I was doing and I responded in sign, ‘listening to music.’ His response was ‘That’s not music! That’s static.’ He tuned the radio to a station and showed me how to hear the difference between static and music—which seems silly and trivial in hindsight, but as a young child with very little music exposure, I had no baseline to work from and I can understand why I thought the static was a (admittedly, strange) form of music.”*

This quote from a pediatric cochlear implant (CI) user (implanted age 3, now age 39), describes the start of his journey to understand music. He played saxophone in lessons and bands from elementary school through college. In his adult life, music remains a valued source of enjoyment, emotional regulation, and social connection. This story of sustained, successful music engagement exceeds outcomes of typical pediatric CI users, who on average have poor pitch and timbre perception and limited music participation (Limb and Roy, 2014; Gfeller et al., 2019b). His experiences are a testament to experience-based plasticity in optimizing the degraded music representation conveyed by cochlear implants (CI).

A deeper understanding of the factors that support successful music training by music-centric (see text footnote 1) pediatric CI can offer fresh perspectives for developing music-based auditory training protocols. This study examined the lived experiences of pediatric CI users who acquired exceptional auditory capabilities through years of intensive music training. The research aims were (a) to characterize the music behaviors and perceptual learning processes of music-centric pediatric CI users, and (b) to identify psychosocial and auditory factors that influence persistence in music-based auditory training as a vehicle for neuroplasticity.

Experience-based plasticity occurs under the following conditions: (a) specificity and salience of the training in relation to the desired neural change; (b) sufficient repetition and intensity of training; (c) a developmentally appropriate progression of skills, and (d) heightened attention and motivation (Kleim and Jones, 2008; Patel, 2011). These conditions abound in long-term instrumental music training, which is an intense, multisensory, and motor experience. Neuroscientists have used music training to study structural brain plasticity in the developing brain in correlation with behavioral changes induced by training (Hyde et al., 2009).

Intrinsic reward is associated with listening to strongly preferred music (Blood and Zatorre, 2001; Patel, 2011); this may enhance perceptual efficiency and promote careful and sustained listening over time (Patel, 2011). The perceptual demands of sustained and heightened attention during music making “fine tune” the auditory system (Meyer et al., 2011; Patel, 2011; Shahin, 2011; Herholz and Zatorre, 2012; Ingvalson and Wong, 2013: Merrett et al., 2013; Olszewska et al., 2021). Research has documented improved auditory working memory, attention, and more rapid spectro-temporal processes in highly trained musicians with normal hearing (Kraus and Chandrasekaran, 2010; Patel, 2011; Shahin, 2011; Olszewska et al., 2021). Torppa and Huotilainen’s (2019) review of music training for children concluded that music training can enhance auditory development in childhood and adolescence.

Studies of normal hearing (NH) persons touting benefits of music training have prompted music-based training for persons with auditory deficits, including CI users (Kraus and Chandrasekaran, 2010; Shahin, 2011; Torppa and Huotilainen, 2019). Reviews of pediatric music training revealed considerable variability in music stimuli, protocols, length of training, and participant characteristics (Gfeller, 2016; Torppa and Huotilainen, 2019; Jiam and Limb, 2020; Shukor et al., 2021). Outcomes were highly variable, and attrition was common. Across studies, training duration of 12 months or longer was required to attain significant and sustained improvement (Torppa and Huotilainen, 2019; Jiam and Limb, 2020; Shukor et al., 2021), which is not surprising, given that auditory perceptual learning requires both stimulus exposure and execution of the task to be learned, along with sufficient amount of practice. Studies of more extensive music-based learning report issues with retention and compliance. While a brief period of training may increase retention rate, it may provide insufficient practice needed for learning, leading to the lack of improved auditory perception (Bissmeyer et al., 2022). Attaining sustained patient compliance in intensive training that supports significant auditory changes is not a trivial challenge for researchers or clinicians (Gfeller, 2016).

What sources of motivation/reward increase intensity and persistence among pediatric CI users, the target population for this study? Developmental considerations are always paramount when pediatric patients are involved. In NH children, music skills develop over many years, but initial engagement typically comprises musical play and exploration in the home (Gfeller, 2016). By middle and high school, participation in lessons and ensembles requires increasingly precise listening and production, sometimes referred to as prestige musicianship.^1^ Disciplined practice, as opposed to music play, can be tedious and frustrating, even for individuals with high auditory sensitivity and accuracy (Yoo, 2020). Many youngsters abandon music lessons within a year (Mazzocchi, 2015).

Psychosocial factors have been identified as influential in music engagement. Initial music instruction and personal practice are often the result of parental encouragement. During adolescence, peer influence increases; music participation is often motivated by the desire for acceptance in a “cool” peer group (Adderley et al., 2003; Bakagiannis and Tarrant, 2006; Lonsdale, 2021). However, social aims may be overwhelmed by the discipline and effort required for more advanced music lessons and ensembles (Mazzocchi, 2015).

Yoo (2020) offered a multifaceted explanation for young people who persist in music ensembles throughout high school, based on the Theory of Planned Behavior (TPB). The Theory of Planned Behavior (Ajzen, 1991, 2012) has been subject to empirical scrutiny in more than 4,200 papers (Bosnjak et al., 2020), and is considered a useful framework for social, behavioral and healthcare research. According to this theory, human behavior is guided by attitude (affective attitude; instrumental attitude), subjective and social norms, and perceived behavioral control. Affective attitude refers to how positively the individual feel toward the behavior (e.g., I love making music); instrumental attitude describes whether the person considers the behavior as beneficial (e.g., making music is good for your brain). Subjective norms include the influence of important people (parents, siblings, teachers, peers), while social norms are informal, mostly unwritten rules that define acceptable actions within a community (e.g., popularity of genres in social media, appropriate uses of music in social situations). Perceived behavioral control includes self-efficacy (e.g., confidence in one’s ability to attain certain goals) and controllability (e.g., perception of ease or difficulty of performing the target behavior). The factors within the theory are inter-related and dynamic. In general, the more favorable each of these factors, the stronger the intent to perform the behavior in question. Specific to sustained music involvement by adolescence, Yoo (2020) found that positive attitude toward music, positive and strong encouragement by teachers, parents and peers, and perception of control and strong self-efficacy were influential.

Positive attitude toward music may also be explained by the intrinsic reward derived from processing music patterns. Listening to personally pleasing music can activate structures in the brain also associated with reward from stimuli such as sex or drugs (Blood and Zatorre, 2001; Patel, 2011). However, given the degraded signal for pitch and timbre (Limb and Roy, 2014; Gfeller and Driscoll, 2020), one can question the extent of intrinsic reward of music listening for pediatric CI users.

Consider the auditory development of NH persons in relation to music. NH listeners have full access to fine structures of pitch and timbre in their daily exposure to a wide range of musical genres and forms. In contrast, CI users whose auditory systems have developed primarily through electric hearing have an “electrodotopic” representation of musical sound. Expanding our notion of musical beauty beyond traditional western harmonies may broaden our understanding of positive music experiences by CI users. Gold et al. (2019) theorize that basic processing of musical patterns engages one’s reward system through a process of updating inaccurate predictions of “what comes next” and validating accurate ones; this process is an important adaptation within daily life. Intrinsic reward from listening is more likely to occur with music that involves a balance of predictability and uncertainty/novelty (entropy), with this balance evolving through on-going exposure to musical patterns (Hargreaves and North, 2011; Gold et al., 2019). For CI users, the pitch, timbral, and amplitude features of music are going to differ from those heard through normal hearing, but those who persist in music training will find patterns, nevertheless.

In order to better understand the auditory learning process, and factors that motivate persistent and intensive music training, this study examined the lived experiences of music-centric pediatric CI users whose musical engagement more nearly resembles professional musicians, including coordination of multisensory and motor systems (as opposed to listening exercises only) (e.g., Kraus and Chandrasekaran, 2010; Shahin, 2011), and who acquired sophisticated auditory perception associated with prestige musicianship. The aims of this study were: (a) to characterize the music behaviors and perceptual learning processes of music-centric pediatric CI users, and (b) to identify psychosocial and auditory factors that motivate or undermine persistence in music-based auditory training; auditory factors included hearing status, sound access, and the auditory characteristics of music encountered.

## 2 Materials and methods

This study examined the perceptual learning processes of music-centric pediatric CI users whose extensive and intensive music training resulted in exceptional auditory skills. We focused on psychosocial and auditory factors as they influenced attention and persistence, both essential to experience-dependent neuroplasticity. Because our aim was to understand these phenomena in depth and within the context of their everyday lives, we chose patient-centered and phenomenological qualitative research approaches as described below.

### 2.1 Approach: patient-centered and phenomenological qualitative research

#### 2.1.1 Patient-centered research

Acknowledges that patients possess extensive knowledge and insights about healthcare through lived experiences (Clancy and Collins, 2010; Sheridan et al., 2017). It complements researcher-driven studies, which tend to focus more on basic science and endpoint outcomes examined through hypothesis testing (Domecq et al., 2014; Pisoni et al., 2017). Stakeholder input has been associated with greater likelihood that the research questions and interpretation will reflect the perspectives and priorities of the target population. Principles of patient-engaged research encourage patient and stakeholder involvement at every stage in research planning, facilitation, and dissemination (Domecq et al., 2014). In the present study, CI users and other stakeholders (e.g., parents, teachers, hearing science professionals) were involved in every stage.

The authors, who conceptualized the study, bring the following experiences and perspectives: the first author has over 30 years of research and clinical experience with cochlear implantation and music. The second author, now an adult, was implanted at age 13. She has been actively involved in music throughout childhood to present time, and is an academic librarian and information specialist. Sixteen stakeholders, all who have experience with pediatric CI users, provided input during focus groups and interviews on recruitment methods and questionnaire content and development. This group included a professor of communication disorders (a SLP whose research includes aural rehabilitation), four clinical audiologists (one is a CI user), three clinical speech language pathologists, four teachers of the deaf (one who is deaf), two parents of CI users, and two CI users (now adults) implanted during adolescence.

#### 2.1.2 Phenomenological qualitative research

focuses on individuals’ lived experiences. It is particularly effective in revealing *patients’ own* experiences of healthcare issues in everyday life, as opposed to categories pre-determined by researchers (Anderson, 2010; Mather et al., 2018). Qualitative methods have been used to examine real-life experiences of CI users, such as listening effort (Hughes et al., 2018), aural rehabilitation (Harris et al., 2016; Glade et al., 2020), and the impact of CIs on music experiences of adult CI users (Bartel et al., 2011; Dritsakis et al., 2017; Gfeller et al., 2019a,c, 2022).

Research questions in qualitative studies are examined through words rather than numbers; data consists of narratives in which participants share their experiences in depth. The participants’ own words are liberally reported within the results (Creswell, 2014; Braun and Clarke, 2019a,b, c). Rather than *a priori* hypothesis testing, research questions are broad and exploratory in nature; researchers generate theories or patterns of meanings from views of the participants (Braun and Clarke, 2019a,b, c; Byrne, 2022). The resulting themes can elucidate previously unidentified issues for future hypothesis testing (Creswell, 2014). For this study, data analyses were conducted using reflexive thematic analysis, which is compatible with the purposes of this study and phenomenological qualitative research (Braun and Clarke, 2019a,b, c; Byrne, 2022).

### 2.2 Participants

Qualitative research utilizes purposive sampling; participants represent particular phenomena relevant to the research questions (Creswell, 2014). Participants included (a) music-centric CI users who had been implanted prior to age 18 and (b) parents of music-centric CI users.

Selection criteria for CI users included: (a) CI implantation prior to age 18 (classified as pediatric patients at the time of implantation) and (b) extensive, successful music making during adolescence. We specified at least 3 years of music making in middle and high school, because ensembles and lessons at that level require more advanced musicianship. We enrolled CI users older than 18 years because they could reflect on their entire childhood and adolescence, and they would have communicative maturity sufficient to write rich narratives essential in qualitative research.

Selection criteria for the parents included: (a) parent of a pediatric CI user implanted prior to age 18, and (b) whose child is/was successfully engaged in music making at least 3 years during adolescence. Enrolling CI users and parents allowed for triangulation (use of multiple data sources), which contributes to methodological rigor (validity) in qualitative research by examining convergence of information (Creswell, 2014). We did not require a one-to-one CI user-parent match because some parents of CI users were deceased or unavailable, and several parents had music-centric children who were not old enough to participate on their own behalf (e.g., currently age 16 or 17).

Tables 1, 2 present auditory profile and music experiences. The pediatric CI users and parents are represented by “CI” and “P” respectively. Tabular information for parents refers to characteristics of their child. P 1 and CI 1 are mother and son; P 5 and CI 5 are father and daughter. The participants grew up in three different countries: C 2 grew up in Singapore and moved to the US, CI 5 and P 5 are from the UK; the other participants all grew up in the US. All use English as their primary language. Participants received their implants and follow-up care from eight different centers, resulting in differences such as access to rehabilitation, or local educational policies.

**TABLE 1:** Auditory profile of CI users (self-reported).

ID	Current age	Hearing loss profile	Hearing aid use?	Age when implanted
CI 1, P 1	39	Congenital bilateral profound sensorineural	Ages 1–2 (reported lack of benefit)	1. 3 years, 11 months 2. 32 years, 3 months
CI 2	34	Congenital bilateral severe/profound sensorineural	HA at 4 months. until bilateral implants	1. 12 years 2. 25 years
CI 3	20	Diagnosed ∼ 1 1/2 years	∼ 1 1/2 years until implants	1. 2 years 2. 2 years
CI 4	25	Post-lingual bilateral severe L: sudden loss around 5 years. age. R: progressive loss, profound by 12 years	From diagnosis until implant use	1. 13 years 2. 18 years
CI5, P5	34	Congenital bilateral, sensorineural severe/profound	12 months to 13 years.	1. 13 years 2. 23 years
CI 6	30	Congenital bilateral severe/profound loss. Residual hearing deteriorated sharply 15 years	From diagnosis, still uses HA in contralateral ear	1. 15 years
P 2	17	Severe to profound bilateral sensorineural	2 months until implanted	1. 11 years 2. 12 years
P 3	16	Pre-lingual profoundly deaf	11 months	1. 17 months 2. 4 years
P 4	25	Diagnosed 10 months	Bilateral HAs after 12 months, used with CIs until 2nd CI	1. 4 years, 10 months 2. 14 years

Characteristics for parent participants (P) refer to their child.

**TABLE 2:** Music behaviors of CI users.

ID	Instrument(s) played	Lessons	Ensembles	Informal music making
CI 1, P 1	Saxophone 5th grade through college, piano-2 years	Ages 10–18	Band ages 9–23 school chorus	Church choir until high school
CI 2	Piano	Age 13–20	NA	NA
CI 3	Piano 5 years, trumpet 3 months., ukulele 3 months., Choroi flute 1 year, violin 8 years	Ages 6–20	Orchestra ages 12–20	With friends 13–20, composing 18–20
CI 4	Trombone	Middle school through MA in college	Band, orchestra	NA
CI 5, P 5	Clarinet, saxophone as adult	Age 7–18, again as adult	Band, wind orchestras ages 8–18	Dance lessons ages 5–8
CI 6	Recorder, 1st grade, brief time with piano, guitar age 15-present	Recorder in class, a few piano lessons, guitar self- taught	Self-formed bands	On-going jamming and composition sessions
P 2	Viola	Ages 10–15	Orchestra ages 10–15	
P 3	Trombone ages 10–15	Starting age 10	Starting age 11, school musical	Electric keyboard ages 8–10
P 4	Trumpet-1 year, guitar, drum, keyboard-most of life	1 year trumpet	Started age 10	Ages 7–18, composing ages 12–15

Music experiences for parent participants (P) refers to those of their child.

Consistent with selection criteria, all CI users had extensive participation in formal music training and/or self-instruction. Two CI users attained college degrees in music; all CI users had attained proficiency in musical skills that exceeded those reported in research (Limb and Roy, 2014; Gfeller and Driscoll, 2020). In relation to research described in the introduction, the experiences of these participants more nearly resembled those of trained musicians, as opposed to persons enrolled in short-term auditory habilitation (Gfeller, 2016).

Sample size is not a straightforward mathematical decision in phenomenological qualitative research (Malterud et al., 2016). Phenomenological studies enroll relatively small samples (e.g., 6–12 participants), with the goal of richness of data and saturation (e.g., same themes expressed repeatedly, no new emerging themes) (Anderson, 2010).

### 2.3 Recruitment

Recruitment of the target population presented a challenge for several reasons: (a) As predicted by stakeholders, accessing pediatric CI users over age 18 can be complicated by their transition to adult CI services; (b) long-term music making by pediatric CI users is relatively rare, and seldom assessed during audiological appointments (Gfeller et al., 2019a,c); these individuals are few in number and difficult to identify; and (c) they are likely to be distributed across many different CI centers and geographic locations.

We recruited this unique population through the following steps: (a) The first author reviewed music involvement of 122 pediatric cases from her center; this yielded 2 potential candidates; and (b) we used snowball sampling (referral from one participant to the next), a technique widely employed in qualitative research when studying hard-to-reach, geographically dispersed populations. This involved contacting five music-centric CIs users known to the authors. We also contacted directors affiliated with (a) seven CI centers whose research includes music, (b) two centers for childhood language, and (c) three advocacy associations for deaf education or serving people with hearing loss. We emailed information regarding the study, with a 2-week follow-up. Those contact persons forwarded email invitations to potential participants. Those who wished to participate responded to the first author.

We used a word-of-mouth approach, rather than posting ads on social media to increase the likelihood that those recruited would truly possess the characteristics of interest. Individuals interested in participation responded to the first author by email, who then emailed that person an invitation to participate, a questionnaire and IRB information. This study was approved by the University of Iowa Human Subjects Institutional Review Board under exempt status; all procedures were conducted in accordance with IRB requirements.

### 2.4 Questionnaire development

Qualitative research methods often use focus groups, in-person interviews, and semi-structured questionnaires (Creswell, 2014). Open-ended questions do not restrict the scope of responses, while also allowing standardization in questions and easy comparison across the dataset (Braun and Clarke, 2019a,b, c).

On-line questionnaires are a reasonable alternative to in-persons methods (e.g., focus groups, interviews) when seeking input from a geographically diverse population. They also have advantages for participants with hearing loss, whose in-person communication may be compromised by factors such as competing talkers and background noise. On-line responses can also reduce transcription errors that can arise from in-person interviews. Prior research indicates that outcomes from on-line inquiries are similar to or even superior to outcomes from in-person focus groups (Tates et al., 2009).

Questionnaire topics emerging from the focus groups included support of influential persons (e.g., family, teachers, peers), environmental influences (e.g., educational system), technology (e.g., CIs), and psychological characteristics of the CI users (e.g., self-efficacy, motivation). These topics formed the basis for initial questionnaire items, which were reviewed by three music-centric CI users implanted prior to age 18. Based upon their input, we made minor changes in wording and added three additional questions to the final questionnaire. The questions encouraged both positive and negative responses. The questionnaires appear in Supplementary Appendix A and B.

### 2.5 Dissemination of questionnaires

Potential participants were sent the questionnaire by email. They were encouraged to: (a) contact the first author with any questions, (b) respond to questions in their own words (no right or wrong answers), (c) avoid any identifying names in their responses, and (d) skip questions they preferred not to answer. Upon return of the questionnaire to the first author, each participant received $25 compensation. We stored returned questionnaires using an alphanumeric ID in a password-protected file. Each respondent’s information formed one case within the complete dataset. Questionnaire responses were copied to word documents, assigned a respondent code, and downloaded to Quirkos software for data analysis.

## 3 Data analysis

Varied methods are used in qualitative research. We chose reflexive thematic analysis as outlined by Braun and Clarke (2019a,b, c) as most suitable for this study. Reflexive thematic analysis is a theoretically flexible interpretative approach consistent with patient-centered and phenomenological approaches. Themes are produced by organizing codes around central concepts that the researcher interprets from the data (Braun and Clarke, 2019a,b, c; Byrne, 2022). An experiential orientation to data interpretation emphasized the meaning and meaningfulness of the data as ascribed by participants. Thematic analyses as described by Braun and Clarke (2019a,b, c) includes six iterative phases as described in Table 3.

**TABLE 3:** Phases and tasks of reflexive thematic analysis (Braun and Clarke, 2019a,b, c).

Phase	Task
Familiarization with dataset	Reading and re-reading data for immersion, familiarization, making notes on initial observations and insights.
Coding	Generating succinct labels (codes) that capture and evoke features relevant to the research questions. Coding involves multiple rounds of coding the entire dataset and collating the codes and relevant data extracts.
Generating initial themes	Examining the codes and collated data to develop broader patterns of meaning (potential themes).
Developing and reviewing themes	Checking candidate themes against coded data and entire dataset looking for a convincing, cohesive story that addresses research questions. This involves splitting, combining, and discarding themes.
Refining, defining, and naming themes	Developing a detailed analysis for each theme and its scope.
Write up	Weaving together analytic narrative and data extracts within the context of existing literature.

We used inductive (data driven) and deductive (informed by extant research, theories) coding. Some items may reflect more than one code; consequently, enumeration of codes reported across themes may exceed the total of coded items. In the iterative process, we examined codes within the context of relevant theories and models, which helped reveal relations among the codes and themes (Braun and Clarke, 2019a,b, c; Byrne, 2022). Items that had either a positive or negative connotation were also coded as having positive (+) or negative (–) valence (e.g., good teachers, + ; bad teachers, -). For this study, research regarding the Theory of Planned Behavior (Ajzen, 1991, 2012; Bosnjak et al., 2020; Yoo, 2020), neural aspects of music training (e.g., Patel, 2011; Gold et al., 2019), and CIs and music perception (e.g., Limb and Roy, 2014; Gfeller and Driscoll, 2020) informed the organization of the codes and themes.

Some approaches to thematic analysis emphasize magnitude coding (frequency, extensiveness) and inter-rater reliability. However, Braun and Clarke consider these approaches inconsistent with the foundations of reflexive thematic analysis. We used sense checking and triangulation to confirm validity and trustworthiness of the data and coding (Braun and Clarke, 2019a,b, c).

## 4 Results

Across the 11 participants, the questionnaire responses yielded a total of 864 coded items, categorized into 50 codes. Data from the CI users and parents yielded 620 and 244 coded items, respectively. The codes were consolidated into and conceptualized through five core themes, listed in Table 4, and discussed below. ID numbers listed along with each quote indicate the source.

**TABLE 4:** Proportion of codes for each theme by group.

Themes	CI Users		Parents	
	% of items	Rank order	% of items	Rank order
1. Musical behaviors	24	2	28.5	1
2. Perceptual learning	15	4	8	4
3. Attitude and perceived behavioral control	33	1	28.5	1
4. Subjective and social norms	16	3	25	2
5. Hearing status and sound access	11	5	10	3

### 4.1 Themes

#### 4.1.1 Music behaviors: music-centric pediatric CI users pursue a variety of challenging formal and informal music behaviors in a sustained, intentional manner. The types and manner of engagement change as a function of maturation, hearing status, background factors, and ongoing social and internal feedback

Theme 1, Music Behaviors comprised 220 of the 864 coded items. The varied forms of music behaviors appear in Table 5. Participants most commonly engaged in music listening, music lessons, playing in ensembles, and playing/practicing instruments. Music making behaviors predominately involved instrumental music as opposed to singing. Two additional categories included intent to engage in music (5%) and description of the musical stimuli (e.g., complexity, rhythmicity, or genre) (11%) experienced during musical behaviors.

**TABLE 5:** Codes for musical behaviors (theme 1).

Code title	% of codes
Listening to music	19.5
Music lessons	11.5
Ensembles	11
Playing an instrument (not in lesson or ensemble)	11
Self-directed training exercises	7
Going to concerts	5
Music games	4
Family music activities	3
Jamming with friends	3
Singing informally (not lessons, choral group)	3
Performing	2
Music as social event	2
Music classes (e.g., elementary general music)	2
Characteristics of music (e.g., structure, genre)	11
Described intention to make music	5

All CI users engaged in music behaviors over many years, but the nature of participation changed as a function of maturation, access to specific experiences, intrinsic (e.g., attitude) and extrinsic (e.g., peer influence) factors. Music engagement in early childhood, described primarily by parents, involved parent-child bonding (e.g., bedtime songs) and playful music exploration. One CI user offered a vivid recollection from childhood:

When I was very small, and before I learned to talk, Mum spent a lot of time singing to me and teaching me pitch and melody. I can remember this so well that I can still recall the intervals she sang—thirds and fifths. [CI 5].

Participants commonly described ambient music as a regular part of family life: “We had radio on at home, music from church …and we listened to music in the car” [P 1]. “We have music playing throughout our home, workout room, garage, and outside patio every day” [P 4]. “Early exposure included listening to my mom and dad’s CD during car rides” [CI 6].

During elementary and middle school, musical behaviors advanced from playful music exploration to more focused playing of instruments and enrollment in formal instruction:

When I was 6–13 …I would just play around with my ukulele, piano, little drums, bells that were laying around …I remember just playing on my harmonica. …I would make up little tunes. …I really liked singing. …I took piano lessons from age six to twelve [CI 3].

The CI users all engaged in formal music instruction during elementary or middle school (see Table 2). “[My son] took trombone lessons at school every morning at 7:30 a.m. before the school bell” [P 3]. All but two enrolled in formal instruction or ensembles through high school.

Two, both guitarists, persisted with formal instructions only briefly, preferring self-instruction or “jamming” with peers. One parent wrote, “He was in the guitar club in high school. He did not have formal training and honestly wouldn’t want it or enjoy it” [P 4]. After receiving his implant, CI 6 “became interested in guitar, I had a couple introductory lessons, but for the most part was self-taught from watching instructional videos and learning guitar tabs.”

During adolescence, music involvement changed in intensity and extent of peer influence:

My involvement with music drastically increased once I was age thirteen. …From ages thirteen to twenty, I got violin instruction from a private teacher …I participated in my high school’s chamber orchestra …From age thirteen to twenty, I played in …youth symphony, where I receive instruction on how to perform like a professional musician …my seriousness about music just grew stronger, the older I got [CI 3].

Informal music listening with peers provided social connection and exposure to diverse music genre. “My friends [and I] would exchange playlists and different artists to listen to. At age thirteen, I would go to my neighbor’s house and sing along to our favorite songs of various different artists” [CI 3]. “When I got my CI …that was a major inflection point when I became much more immersed in the world of music, branching out to more diverse variety of both older and current artists, attending more concerts, and learning guitar and joining a band” [CI 6].

Participants characterized music engagement as dynamic and varied: “I think experimenting with different means of engaging with music …is important” [CI 6]. “My hearing has changed so much through my life, and so has my ability and experience with music. I’ve always loved listening to music from eclectic and varied genres” [CI 5]. Experimentation with various instruments helped reveal the most satisfying music options: “I did jump instruments a lot before I finally settled down with the violin as my primary instrument. I have played piano for 5 years, trumpet for 3 months, ukulele for 3 months, and Choroi [flute]” [CI 3].

Participants described most music experiences in positive terms, but specific tasks presented unattainable challenges. For example, CI 1 participated in choir for several years; his parents’ policy was to stay with a music class for at least a semester before quitting: “It was more difficult for me to match pitch with my voice than with an instrument like a piano or a saxophone …My singing voice was not always right, so I felt a degree of embarrassment from that, and after I was allowed to not select chorus, I was relieved!” [CI 1]. In contrast, he participated in bands throughout college.

Music remains a valued part of life beyond adolescence, though personal goals and motivation differed among participants: “I still connect with people for on-off jam sessions. I’ve also lent my guitar playing to recordings that my friends and colleagues have put out” [CI 6]. “I’ve done all the exams I want to do, then I stopped making music for a few years, and now I have come back with more of a beginner’s mindset and a willingness to relearn things” [CI 5]. “I enjoyed listening to music (still do) and it’s a valuable way for me to allow myself an escape from everyday listening that doesn’t require me to turn my implants off” [CI 1]. One parent noted that her adult son, “still continues to teach himself to play the guitar, drums and keyboard, make music, goes to concerts and has many music apps” [P 4].

While most of the CI users who had reached adulthood engaged in music for personal enrichment and social connection, two completed college degrees in music, majoring in violin (Associate of Arts Degree) [CI 3] and trombone (Master of Music) [CI 4]. Collegiate music studies demand extraordinary listening sensitivity and commitment: “My education included advanced music theory, sight reading… analyzing orchestral and symphony works, composition… recitals, taking violin lessons, and performing in a symphony orchestra.” [CI3] Both didactic (e.g., music theory) and performances requirements (e.g., lessons, ensembles) in most collegiate music curricula involve extensive and demanding listening skills.

The extensiveness of music engagement among these CI users is remarkable, given the degraded music signal conveyed by the CI. The following theme focuses on the learning process through which these CI users sought musical meaning and attained precision musicianship.

#### 4.1.2 The development of perceptual skills required for prestige musicianship comprises a lengthy dynamic feedback loop of exploring and responding to diverse musical stimuli within changing contextual circumstances. This learning process is moderated by background factors (e.g., cognitive processing), hearing status, intrinsic characteristics, and social influences

Theme 2 (110 coded items) focuses on the process of auditory skill development while engaged in the musical behaviors described in Theme 1 (see section “4.1.1 Music behaviors: music-centric pediatric CI users pursue a variety of challenging formal and informal music behaviors in a sustained, intentional manner. The types and manner of engagement change as a function of maturation, hearing status, background factors, and ongoing social and internal feedback.”). Attentive music listening and playing was an integral part of everyday life and occurred over years. As documented in Theme 1, music engagement included varied experiences (listening, lessons, ensembles, etc.) involving multiple systems (e.g., auditory, motor, visual) through breadth and depth of involvement.

CI users commonly described this process as “learning to listen,” requiring intentionality and persistence; they actively pursued challenging listening experiences. Repeated exposure to musical stimuli resulted in reduced perceptual entropy and increased predictability in musical stimuli: “Most people need time to decipher and learn how to hear again” [CI 4]. As one CI user stated, “It’s not going to be like flipping on a light switch… it does take time and sometimes my brain doesn’t want to cooperate…” “[playing music] requires a lot of work and exposure to the things you want to understand. The more exposure you get yourself, the more you will understand how this sound should be” [CI 1]. “It’s definitely possible to enjoy music with a CI; keep listening and you will be able to appreciate it more and more. Don’t give up early; it’s a process” [CI 2].

One CI user “initially hated music through my implant… I listened to quite complex and layered music, which was a real challenge, but which really helped my musical ‘ear’ to develop, so to speak” [CI 5]. The CI users recommended varied and focused experimentation: “Try to listen to everything. It will be tiring and missing things is part of the process, but at the end of the day, listening more leads to hearing more” [CI 4]. “I would advise lots of practice and active listening—so playing an instrument, choosing new and challenging… I think experimenting with different means of engaging with music is also important” [CI 6]. “Listening to music and learning how to play an instrument definitely made me hear much better with CIs. I encourage everybody who gets a CI to learn how to play an instrument” [CI 2].

CI users developed listening strategies within their processes: “In marching band in college… I grew to identify the sound of other saxophones to help myself stay in tune and in sync with them” [CI 1]… “Paying close attention to my stand partners and making sure I am in time with them” [CI 3]. “Using a tuner helped with learning what certain notes sounded like. With practice over time, it became easier to identify concert F” [CI 1]. Other strategies included use of muscle memory, vibrotactile input, attentively listening to different pitches at the piano, and watching the lyrics on music videos.

The participants described structure, habits, and realistic expectations as keys to success: “I learned the lesson that small steps are the only way things happen… I learned all the basics… in small steps over the years, which made me so much better as a player” [CI 3]. “Be persistent and consistent with practicing and learning any new instrument. Dedicate time every day to devote to practicing” [P 2]. “I found that every day of playing the piano and listening to music in the early years of using my implant helped to hear better with it, so I kept at it because I also wanted to hear better” [CI 2]. “I think getting that music exposure while going through auditory training helped a lot and it’s clearly had a lasting impact” [CI 6]. “‘[My teacher said] why don’t we practice good habits to… get the most out of what you have?’ From that point on, I approached every challenge from the angle of ‘what habits do I need to accomplish this?’ rather than, ‘I can’t so I won’t” [CI 4].

The formation of habits requires motivation and persistence in the face of incremental progress and frustration. The following theme focuses on personal characteristics that undergirded motivation and persistence.

#### 4.1.3 Attitude and perceived behavioral control: sustained music learning is associated with self-efficacy, positive attitude, and personal identity

(a) CI users who persist with music have strong self-efficacy and perceived behavioral control; they enjoy and feel capable at handling challenges.

(b) Persistence is supported by positive affective attitude toward music and instrumental attitude–perceiving that music is beneficial to other functional areas (e.g., cognition, emotional wellbeing).

(c) Music-centric CI users consider music an important part of personal identity.

Theme 3 comprised 289 of the 864 coded items. This theme, informed by the Theory of Planned Behavior (Ajzen, 2012; Yoo, 2020), addressed intrinsic factors that influence behavioral intent.

Self-efficacy is context specific and associated with perceived behavioral control. It is characterized by belief in one’s capabilities to organize and execute the course of action required to manage situations. Considered a personality characteristic, self-efficacy can also be enhanced through modeling of self-efficacious behaviors by parents and teachers, success in past experiences, and positive emotional states. Self-efficacious people are self-motivated and persist in the face of adversity (Bandura, 2010). They are more likely to see challenges as an opportunity as opposed to a barrier, and self-advocate in difficult circumstances (Gfeller et al., 2019a). In the context of music behaviors, these CI users showed strong self-efficacy, self-advocacy, and perceived behavioral control in music, despite the challenges associated with music and electric hearing.

Quotes characterizing self-efficacy and perseverance included: “For me, one of the most important parts of my journey with music and the CI was to stick with it even when I wasn’t hearing it as optimally as I could” [CI 6]. “It is really important to persevere because getting an implant, and having that implant tuned, can be really disruptive. You have to keep getting over barriers, but having the end goal in sight is what makes it worthwhile—the pleasure of enjoying music” [CI 5]. “A huge belief that helped me stay in music when it was hard music and felt hard facing it, was small steps… small steps, made me feel like it was possible to do music when it was demanding” [CI 3].

One parent spoke of his daughter’s “extraordinary focus and determination… a very talented, resourceful and single-minded child… [who] never accepts ‘you can’t”’ [P 5]. “Making music presents a challenge. When I get in the flow and create music that sounds good to me, it’s wonderful. Other times it’s frustrating… it is a hearing challenge as much as a musical one. But I continue… because it is fun” [CI 5]. “Always self-advocate… in rehearsal, with friends listening to music, or in a venue listening to a concert” [CI 3]. “Really advocate for yourself so you can get what you need and get the support you need… [my son] created a slide show about his hearing loss and CI so his teacher understands his hearing loss and how his devices work so they can know how to help better” [P 2].

Another intrinsic characteristic informed by the Theory of Planned Behavior (Ajzen, 2012; Yoo, 2020) is attitude: affective attitude toward the behavior, and instrumental attitude–the belief that the behavior will have positive benefits. Individuals who have a favorable attitude toward the target behavior have stronger behavioral intent.

Parents commented, “Her enjoyment of music gave her a great sense of achievement… we know that it brings a lot of pleasure” [P 5]. “He always loved listening to music” [P 3].

I believe he was never disappointed or felt that he wasn’t “hearing” music like his family, friends, or other people. He only knew what music sounded like to him and he enjoys it immensely… He always had positive interactions with music… He was always determined to enjoy music, either alone or with friends [P 4].

Several CI users considered strong passion a key aspect to sustained involvement:

Overall, I think the baseline interest or passion has to be there to some degree, otherwise it will be tough to put in the time it takes to adapt to hearing music with a CI… When I got the implant… re-learning how to hear with the CI [compared with hearing aids] and not everything sounded great. But I heard enough new sounds in music that excited me to the point that I wanted to pursue it, even when the sound perception wasn’t great… if one genre of music isn’t enjoyable, try another until you find what you like listening to [CI 6].

A few participants described psychological benefits of music: “[Music] helps me tap into emotions” [CI 3]. “Just like anyone else who listens to music, I get relaxation and comfort for a moment. For the duration of the music, I don’t have to worry about anything else and can just focus on enjoyment” [CI 2]. “Music helps with reducing distractions… it enables me to focus on my work… Making music for pleasure… brings me peace and allows me to express emotions or reflect on these emotions in a healthy way… a valuable path for my mental health” [CI 1].

Two CI users described physical benefits: “I really love dancing… music… allows me to feel deeply alive inside myself and gain more awareness of my body” [CI 3]. “When I work out, I like music with heavy beats as they help keep me focused… It also helps with moderating my speed and intensity” [CI 1].

Several parents discussed benefits of music. One parent, in completing the questionnaire, asked her son how music affected his life. She quoted his response: “It’s good for my soul. It helps me focus many ways. It’s personal” [P 3].

Having access to sounds and music has had such a positive effect on my son’s life. I’ve thought about it many times over the years… what if we didn’t give him access to… music through HAs and CIs… how much enjoyment, relaxation, friendships and pleasure he would have missed out on [P 4].

Nine of the 11 participants described music as part of personal identity: “I’m glad I had the musical experiences I had growing up… they have shaped me in ways… I’m happy about” [CI 1]. “Playing music and listening to music has been a huge and integral part of my life since I was small” [CI 5]. “I deeply acknowledge that it can feel hard sometimes being the only deaf person in the room [rehearsal space]… But every single [person] brings a unique gift… There is always room for you” [CI 3].

One CI user described a very personal relationship with her violin:

I want to share this really cool thing… My violin [and I] are connected in a really deep way. My violin always shows me what is happening with me. If I show up to my lesson tense and grumpy, it shows up in my playing. And I notice that it is a message to give myself greater care… I always feel like my violin tells me the right thing I need to hear or pay closer attention to [CI 3].

Several parents reflected on music and personal identity: “I can’t imagine him without his implants… Music is part of him… His love for music never stops… passion/natural love for music [motivated him]” [P 3]. “He was very proud to be a member of [the college marching band]” [P 1].

One parent remarked on how music continues to reflect her son’s identity into adulthood:

My son has a baby girl now and he sings and plays classic rock music with her. *Honky Tonk Women* by the Rolling Stones is their favorite and she will always calm down hearing this song. My son also has a… chocolate lab dog and his name is Zeppelin, after Led Zeppelin band. Music definitely has been a positive experience in his life, and I couldn’t imagine his life without his CIs and music [P 4].

This final vignette addresses not only self-identity but also social connections through music, which is the focus of the following section.

#### 4.1.4 Subjective and social norms: sustained intention in musical behaviors is influenced by family, teachers, peers, and social norms. The strength of specific influences changes as a function of age, situational circumstances, and quality of interactions

Theme 4 comprised 162 of the 864 coded items. According to the Theory of Planned Behavior (Yoo, 2020), behavioral intention is influenced by extrinsic factors of subjective and social norms. Subjective norms are attitudes of important persons (e.g., parents, teachers, peers) toward the behavior. Social norms refer to the customary codes of behavior by groups or larger cultural context. The most prevalent coding categories were parents (54 items), peers (42 items), teachers (34 items) and social norms (e.g., popular music trends) (17 items). Hearing health professionals comprised six coded items: five described support from deaf education teachers; audiologists were mentioned only once as uninvolved in support of auditory training.

Parents had primary responsibility for access to informal and formal music experiences, especially prior to adolescence. Parents encouraged and facilitated access to music in daily life, considering music to be a valuable experience and choosing to direct family resources toward music experiences such as lessons, concerts, instruments, and recordings. “[She was] brought up in a music-loving family. One parent was an early years teacher focused on maximizing sensory inputs including singing, musical toys, etc.” [P 5]. “My dad played music for me when I was two, and saw that I was enjoying it… ever since then, my dad has always encouraged me to pursue music if I wanted to. And I did” [CI 3]. “His dad played some music instruments in high school band, so… we have guitars, drums, and keyboards in our home. His dad loves music and we attend concerts together… My son and husband built his own queen size bed when he was around 12-years old and put a stereo system and built speakers into the headboard and frame so he could listen to music in his bed” [P 4].

Parents facilitated enrollment in music lessons: “My parents encouraged me to play the clarinet after I expressed an interest in learning an instrument” [CI]. “I’m always super thankful to my mom and dad for supporting me with my music, taking me to my symphony rehearsals and violin lessons.” [CI 3] Access to music included overcoming technical problems with the CI: “There was once he was driving to a concert in Chicago with his best friend. The one CI battery died and I drove an hour to meet up with him to give him another charged battery. He didn’t want to go to the concert with only one CI” [P 4].

All the parents expressed the importance of music exploration as illustrated in these quotes: “Let the child try everything, have music playing, watch musicals, go to the theater, sing in choir, and play in band. You never know what they can and cannot do unless you try” [P 1]. “My parents wanted me to explore whatever I wanted and to fully support whatever I chose” [CI 3]. “We encourage him to develop his own gifts. Nothing is impossible” [P 3]. “Always go for it and anything else that stretches the brain’s new connections” [P 5].

In addition to providing access to music experiences, these parents modeled positive attitudes toward music involvement: “Music has always brought enjoyment and relaxation into our home, so we’ve always had a positive attitude toward it” [P4]. “Everyone in my family was very encouraging” [CI 6]. “We always played music on the radio and stressed the importance plus benefit of learning an instrument” [P 2].

We had an enthusiastic and supportive home environment where older sibling played the flute and all enjoyed regular theatre and concert visits… It was obvious… how much [she] enjoyed listening to music and we loved seeing her take part in this typical teenager “rite of passage”… It’s all about enthusiasm, enjoyment, and a supportive framework [P 5].

Parents also influenced self-efficacy: “My mom has always been supportive and I try to succeed in everything I do to not let her down. My dad… has always been skeptical. So I try to succeed in everything I do to not prove him right” [CI 4]. “Sometimes when I feel down and don’t want to practice my music, my mom would notice and she always would remind me of the core reason why I love music, which is the emotionality of music” [CI 3]. “Once the kids started something, they had to finish the year or season” [P 1].

Within the family unit, siblings also influenced music learning: “I wanted to be like them [older sibling]… I would listen to the same stuff [my older sibling] listened to, to get an understanding of what music was at an early age while I was learning how to listen to music” [CI 1]. “[His] older brother was also in choir and band… His older brother played for a [different college] marching band. He was very competitive” [P 1]. “I wanted to because my older (hearing) sister played the flute” [CI 5].

The influence of peers and social norms strengthened during adolescence. The CI users socialized through music and engaged in the sorts of music behaviors one would see among typically hearing adolescents (Adderley et al., 2003; Laiho, 2004; Bakagiannis and Tarrant, 2006):

“Music was a huge part of my peer group as a teenager… we used to go to live gigs together and crowd into bedrooms and listen to mix tapes… I wanted to fit into quite a ‘cool crowd’… And the music was a huge part of that… Music became a big part of my identity” [CI5]. “High school saw a new friendship group and a new interest in rock and metal music!” [P 5].

“I shared and listened to music together with 2 good friends between the ages of 16–18” [CI 2]. “Music was shared and discussed as a kind of social currency around what was popular and trending at the time… [after the CI] I enjoyed having deeper conversations about music and artists and seeking out new music” [CI 6]. “When I got to high school, the kids in my friend group were extremely involved in music, so I gravitated toward trying to do what they were doing… I was pushed into the music arena by friends, and I’m grateful every day for it… My friends… helped me feel welcomed” [CI 4].

“I think it is a really powerful thing to have friends who want to share playlists and bond over music, because there is a deeper opportunity to make connections with other people and see yourself reflected in the music you share… My friends really were the ones that strengthened my mainstream/alt pop exposure and influence. I have many thanks to them for doing that because it did change me as a person” [CI 3].

Peer influence continued into adulthood: “Now [as an adult], I use a group of friends who are very heavily invested in music to get information about new songs or groups I should listen to. As a result, I have a very wide preference of music listening taste” [CI 1]. “I have one very close friend who deeply impacted me with an introduction to the kind of music at age eighteen that I listen to today… All of my current listening music is all music and artists that she has introduced me to” [CI 3]. “Some of my closest friendships to this day started over mutual appreciations for music” [CI 6].

Peers also supported perceptual skill development: “My best friend would print the lyrics off and help me to follow them by pointing with her finger in time to the music” [CI 5].

[My friends] would spend extra time to help me hear and understand things. [My friend] “would sit at a piano and help me learn to hear sequences and different pitches I was hearing wrong; she helped me listen better to musicians around me (it is an art to hear in the moment); and more than any of these things, she always felt I could hear a little more if I tried. That was the real ticket” [CI4].

Two parents of CI users currently in high school specified that although their sons enjoyed making or listening to music with peers, they retained a strong personal preference in music: “[my child] made his own choices and doesn’t really let others’ choices influence his decisions” [P 2]. “[He] knows what he likes” [P 3].

Music teachers were a powerful influence whether positive or negative: “In grade school band, there were two teachers. One was very patient and encouraged him to continue in band. The other was not patient, not caring and did not encourage him or help him” [P 4]. Parents and CI users emphasized the importance of finding good teachers who could encourage as well as instill careful listening and good practice habits.

“His private teacher has always been motivating… and given him support and positive feedback” [P 2]. “[Both teachers] always encouraged him. We were blessed to be around people like them” [P 3]. “Both my piano teachers were encouraging; never felt that my deafness or implant was a barrier to learning music” [CI 2]. “My biggest thanks to people that helped me feel successful and very encouraged to be a musician is to my violin teacher” [CI 3]. “We had him attend [an aural school] where they encourage playing music from toddler to 8 years old before mainstreaming in a public school” [P 3].

Teachers were also important in fostering careful listening, setting challenges, and establishing good practice habits. “His private teacher was instrumental in teaching good habits and proper technique. This made learning music more enjoyable” [P 2].

Schools provided excellent hearing-impaired support including… excellent music teachers… [they] encouraged making the most of residual hearing… (this was not mentioned by the Audiology Department!) and reinforcing hearing… by rewards… our child made great progress. [They] had a music group, which was great at using music to incentivize careful listening [P 5].

CI users considered the expectations set by their teachers an essential component in auditory skill development: “I wasn’t really treated any differently by music teachers in primary and secondary school” [CI 5]. “They would make me try to differentiate pitch, describe melody, describe rhythm, describe music dynamics etc.” [CI 2]. “[My teacher] made an impact I still feel reverberations from… [He] knows what drives success: habits” [CI 4]. “I feel really special to have a teacher that honored… my process as a musician and supported me in a really kind way. She also helped encourage me to persevere when things felt hard in life or with my instrument” [CI 3].

While these first three themes could easily apply to most any young musician, the final theme highlights the unique auditory challenges faced by CI users in music learning.

#### 4.1.5 Hearing status and sound access: the CI and other technology are valued for providing sound access, but processing limitations and poor acoustic circumstances require on-going adjustment and acceptance

Theme 5, which focuses on hearing status and sound access, included the smallest number of coded items: 93 out of 846. The narratives described their auditory profiles, benefits or limitations of hearing devices, necessary adjustments, and the desire for improved CI technology.

Four of the CI users received their first implants before the age of five (implanted between 17 and 58 months). Five used hearing aids for many years before receiving their first implants between 11 and 15 years old.^2^ Those implanted after age 11 contributed a larger proportion of items regarding the impact of their hearing device (15% of all codes) than those implanted prior to age 5 (7% of all codes).

Although CIs convey degraded representation of musical sounds, the CI users implanted after 11 engaged with music more enthusiastically *after* implantation. Though acclimatization was necessary, they described the CI as offering greater sound access than hearing aids, which opened new musical opportunities. “[My son] said it was easier to hear music with his implant than his hearing aids” [P 2]. CI 5, who started music lessons at age 7 with her hearing aids, commented on the transition to her CIs: “When I got my CI, this became my ‘ear’… After I had my first implant aged 13, I was able to experiment a bit with connectivity… (direct input cables), and these were quite good, although I initially hated music through my implant.” [CI 5] CI 4, who used HAs until age 13, emphasized the benefit of bilateral implantation: “It wasn’t until I went bilateral that I felt confident in my immediate environment.”

Two CI users implanted at age 12 and 15, respectively did not engage with music to any extent until after their first implant: “No involvement with music before cochlear implantation; after cochlear implantation, began teaching myself how to play the piano, downloading/listening to music, listening to MTV, sharing music on portable music devices with friends” [CI 2].

I never engaged much with music until I got my cochlear implant, when I was about 15 years old. “While going through auditory training, I slowly discovered that the combination of the CI in my right ear and hearing aids in my left ear added a clarity to music that I never heard with my hearing aids alone… from that point on I became an avid listener of many different genres of music and began playing the guitar—and I haven’t looked back since!” [CI 6].

While all the participants expressed gratitude for better access to sound through the CI, several also described limitations in sound quality and perceptual accuracy: “Whenever my implants get reprogrammed, it gets weird playing my instrument because my brain has not gotten used to the new programming because everything sounds like a warble instead of actual sound” [CI 3].

As great as it is that the CI has unlocked a new sound range for me, it can still sound digital/tinny at times and it’s tough to discern individual instruments when multiple parts are being played at once. I also find that the bass guitar is one instrument I can almost never make out… all that said, I’m still grateful for what it allows me to do and hear today [CI 6].

Some musical tasks remained more resistant to improvement, despite training:

I am unable to discriminate pitch well… it was hard to know what specific notes were being played. I could approximate the melody but never perfectly. I am unable to pick out specific instruments or listen to lyrics well in music sometimes since I listen to it as a whole. I have pretty much accepted it, which doesn’t lower my enjoyment, but it does make certain genres difficult to listen to [CI 2].

The extent to which CIs were beneficial also depended on the listening environment: “The most annoying experiences took place outside or in big, boomy rooms like a gym or multi-purpose rooms” [CI 4].

None of the participants considered special processing features beneficial, but they expressed hope for more effective technical options in the future: “I very specifically did not want ‘smart’ features with my MAPs because I found that they tend to dim the amplitude of whatever I was listening to” [CI 2]. “I have experimented with different CI programs… for the most part I leave the CI on my normal everyday setting when I play music… this is an area that I do hope to see advance in the future” [CI 6].

It is a shame a “music map” is not offered to CI recipients. We grow up in this muted, sound-filtered world without any idea of how to filter it ourselves. I only ever use the non-filtering map in my implants. The complexity of sound from letting things blend is natural… most people need time to decipher and learn how to hear again [CI 4].

Low battery life was a source of frustration: “I do remember one situation that was super frustrating. I was sitting down in symphony rehearsal performing… a few minutes in, my cochlear implant batteries completely died. It made me feel really overwhelmed and upset… having implants die mid performance… is very limiting for the satisfaction of playing” [CI 3]. She realized the need to prevent future battery crises: “What has really helped me. is having a fully charged spare of batteries always on the go in my purse” [CI 3].

Several of the participants mentioned benefits of assistive technology or apps. Two mentioned that streaming of lyrics helps with song recognition or understanding lyrics against the accompaniment: “The rise of lyrics… with streaming apps has made a massive difference in my listening habits” [CI 1].

Other beneficial technology included Roger pens to hear music teacher instruction, iPhones, Bluetooth, and closed captioning. Three CI users mentioned tuning apps as beneficial for playing in ensembles: “I use my cleartune app for tuning my violin. I love how the little tuning meter shows how close or far you are from being in tune” [CI 3].

An important aspect of qualitative research is examining the relations among codes and themes and how they relate to relevant research and theories. That is the focus of the following section.

### 4.2 Relations among the themes as informed by relevant research and theories

**FIGURE 1:**
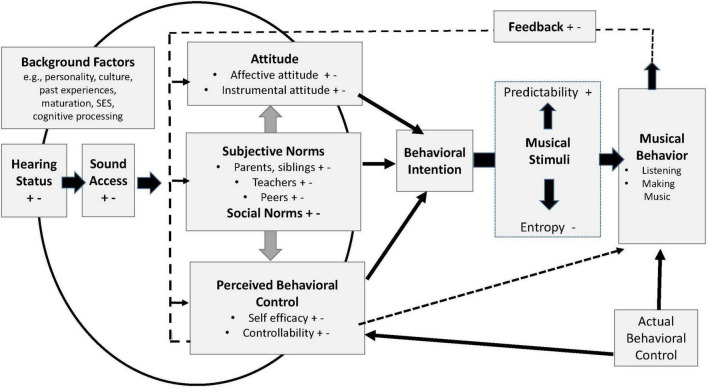
Model of music-based learning for pediatric cochlear implant users. +, positive influences; -, negative influences. Hearing status = hearing history, auditory profile, hearing devices. Sound access = functional listening as influenced by listening circumstances, device benefit. SES = Socioeconomic status.

For this study, the Theory of Planned Behavior (Ajzen, 1991, 2012; Bosnjak et al., 2020; Yoo, 2020) provided a foundation for conceptualizing music learning. However, this theory does not address auditory factors of CI users, the diversity or varied complexity of musical stimuli, background factors, (e.g., Socio-economic status (SES)), or the ongoing changes in perceived predictability and entropy in the music stimuli (Gold et al., 2019). Those factors were integrated into a model, which conceptualizes the five themes: Model of Music-Based Learning for Pediatric Cochlear Implant Recipients (MMBL-PCIR) (see Figure 1). Together, the five themes present perceptual learning as a lengthy dynamic process involving diverse music behaviors, which are influenced by intrinsic and extrinsic factors.

Theme 1 is represented by components of Musical Behaviors and the Musical Stimuli encountered; these appear in the right half of Figure 1. Theme 3, which focuses on intrinsic factors, is represented by Attitude, Perceived Behavioral Control, and intrinsic characteristics within Background Factors (e.g., personality and cognitive processing). Subjective Norms (parents, teachers, peers), Social Norms, and Background Factors such as culture and socioeconomic status (SES, a family’s economic access to resources and social position), make up the extrinsic factors of Theme 4. The components of Themes 3 and 4 are interactive. For example, a serious discrepancy between actual and perceived behavioral control can undermine positive attitude and self-efficacy; conversely, a supportive teacher can enhance positive attitude and self-efficacy. Families with higher SES can offer greater exposure to music lessons and concerts.

Theme 5 is represented by hearing status and sound access. Hearing status refers to hearing history, auditory profile, and hearing devices; sound access references functional listening as influenced by listening circumstances and device benefit.

Theme 2, the process of perceptual learning, involves the dynamic interaction of all the components in the model. In the model, those interactions are represented by arrows and lines intersecting or connecting components, and indicating on-going feedback from musical behaviors that influences psychosocial factors; these influence behavioral intent. The curved line intersecting Hearing Status and Background Factors represents the influence of those factors on Attitude, Subjective Norms, and Perceived Behavioral Control. One example would be a dead CI battery, which undercuts access to the verbal encouragement from teachers and musical stimuli, which in turn diminishes perceived behavioral control and positive attitude. Another example is that efficient cognitive processing can facilitate processing of musical patterns and thus, increased sense of control.

The strength of behavioral intent varies depending how positive or negative the various factors are (represented in Figure 1 by + and–symbols). The more positive factors, the stronger the behavioral intention is likely to be. In relation to musical stimuli, the ease of processing and enjoyment of music will vary as a result of prior familiarity with a given style or composition, or repeated exposure to a specific piece of music. Positive valence (+) is associated with greater predictability, and negative valence (-) is associated with greater entropy; the black arrows represent shifting perception of entropy to predictability within the musical stimuli over time.

By applying this model to music learning, parents, teachers, clinicians, and researchers can analyze and address factors that foster positive learning conditions. While the extensiveness and intensity of these lived experiences are difficult to replicate in short-term controlled experiments of music-based training, researchers should consider intrinsic and extrinsic sources of reward in their choice of musical stimuli and protocol characteristics that could motivate attentive persistence. As these CI users indicated, their commitment to music listening was not solely a product of the signal from their hearing devices. To encourage optimal motivation and training intensity, a research protocol for adolescents might include peer interactions, socially meaningful and sufficiently diverse musical stimuli, and include opportunities for behavioral control. Parents and teachers should be enrolled into the aims of the study. Those factors that cannot be readily manipulated (e.g., personality or cognitive characteristics) could be measured (e.g., measures of self-efficacy or pattern recognition) to better understand variance across participants.

## 5 Discussion

The lived experiences of these CI recipients indicate that music learning is a complex process involving a host of influential psychosocial and auditory variables; this includes parents, teachers and peers, social and emotional rewards, and varied and meaningful musical stimuli. The musical experiences of these CI users are remarkably similar to those of typically hearing youth (Adderley et al., 2003; Bakagiannis and Tarrant, 2006; Bullerjahn et al., 2020; Yoo, 2020). These findings are also consistent with prior quantitative studies of children with CIs reporting parental support as highly influential in music engagement (Gfeller et al., 2012, 2019b; Driscoll et al., 2015). Consequently, one step toward facilitating successful pediatric music training (clinical or research) should be involvement of parental support and modeling self-efficacy (Smith and West, 2006). These CI users also gravitated toward music for social affiliation and as part of personal identity (e.g., Laiho, 2004; Lonsdale, 2021); thus, for adolescents, training that also includes peers may have greater social currency.

An important difference between the musical engagement of these CI users and some music training programs (for review, see Gfeller, 2016) is playing of instruments over many years, in contrast with brief, online listening exercises. These CI users engaged in intense, multisensory, and motor experiences. Prior studies of music training indicate that the pairing of auditory input with tactile and motor input can strengthen neural connections (Hyde et al., 2009).

Normal hearing individuals often listen to music for mood regulation and pleasure (Gfeller, 2008; Sloboda, 2010; Bullerjahn et al., 2020). Despite the CI’s degraded representation of pitch and timbre, these CI users commented extensively on emotional benefits and pleasure derived from music. This contrasts with group data from pediatric CI users who experienced less satisfactory enjoyment and emotional response to music (Whipple et al., 2015; Gfeller et al., 2019b). This suggests that more intensive music training can enhance emotional as well as perceptual access to music.

These CI users reported definite musical preferences, the influence of social norms, benefits of varied music, and trial and error. These were all integral to active listening and exploration. Obviously, some research questions demand highly controlled and decontextualized stimuli, but the perspectives of these CI users suggest that whenever possible, intrinsic motivational aspects of musical choices should be a high priority. Stimuli should also be chosen that offers a reasonable yet challenging balance of perceptual entropy and predictability to enhance motivation and perceived behavioral control (Hargreaves and North, 2011; Gold et al., 2019). The most theoretically ideal training stimuli will not benefit a listener who is not motivated to attend or persist.

Given the technical limitations of the CI in conveying pitch and timbre (e.g., Looi et al., 2012), we were surprised by the small number of codes specific to CI technology, especially among CI users implanted prior to five years. Perhaps early implanted CI users consider electric hearing as a more “normal” part of life than later-implanted individuals do. Also of interest was the enthusiastic engagement with music for several CI users following implantation. The enthusiasm of this cohort for music seems consistent with a recent study comparing prelingually deaf CI users with post-lingual adult CI users; prelingually deaf young people found music more enjoyable than individuals who had grown up with music (Fuller et al., 2019).

The limited focus on device limitations may reflect a broader conceptualization of meaning in music. Musical meaning is a mental creation of the listener, who imposes organization on and assigns meaning to patterns of acoustic events; this meaning is derived from all prior listening experiences, especially the musical grammars of one’s own culture. Music components (e.g., scale intervals, choices of timbre, importance of pitch, etc.) vary from one culture to the next (Gfeller, 2008). One could argue that CI users whose auditory development resulted from electric hearing comprise a musical sub-culture.

Consider the words of CI 2: “I am unable to discriminate pitch well.^3^ … I could approximate the melody but never perfectly… I am unable to pick out specific instruments. *I listen to it as a whole. I have pretty much accepted it, which doesn’t lower my enjoyment*.” If one based his music outcomes on pitch perception alone, his results might be characterized as poor to average. However, he derives enormous pleasure and meaning from music; furthermore, his public performances of advanced repertoire (e.g., Chopin etudes) reveal subtle interpretation and sophisticated musicality (Personal observation by the first author).

Even for normal hearing individuals, there is no “gold standard” for what constitutes beautiful and thus rewarding music (Gfeller, 2008; Hargreaves and North, 2011). Musical beauty is an elusive construct. Genres such as hard rock or alternative jazz use growling or distorted vocals or instrumentals, and “bent” intervals as important stylistic features. Genres such as rap and some ethnic music are predominately rhythmic patterns; melodic features are less important. Furthermore, when people with normal hearing listen to unfamiliar musical styles/forms, they may access the sound easily, but have difficulty understanding and deriving meaning from the patterns (Gfeller, 2008). The diversity of musical tastes, cultures, and past music experiences [which do influence processing, entropy, and predictability (Gold et al., 2019)] presents a daunting challenge for researchers or clinicians when selecting musical stimuli that has motivational value, or in choosing relevant measures of training benefit (e.g., measures of sound quality, pattern recognition).

The data from this study reveal interesting similarities and differences between pediatric and adult CI users who are music-centric (Gfeller et al., 2019a,c, 2022). Both groups demonstrated high self-efficacy, perceived behavioral control, and positive attitudes toward music. Both groups had strong intentionality and intently pursued challenging music experiences. Embracing a challenge and tolerance for frustration were important aspects of their successes (Bullerjahn et al., 2020; Wang and Jiang, 2022). However, subjective and social norms played a greater role in lives of pediatric CI users. For these pediatric CI users, access to music experiences was controlled to a considerable extent by parents (Gfeller et al., 2019b), and peer influence was particularly important during adolescence. In addition, adults with normal hearing during childhood and implanted well into adulthood were more likely to use their memories of musical sounds and repertoire to complement the degraded signal of music through the CI (Gfeller et al., 2019a,c). Only one CI user in this cohort described drawing upon a specific memory of musical sounds from childhood: the sound of her mother singing thirds and fifths during her preschool years.

For both adult and pediatric CI users, repeated exposure to music resulted in a shifting perceptual balance between entropy and predictability of musical stimuli (Gfeller et al., 2003; Hargreaves and North, 2011), though actual percepts differ markedly from those of NH people. Research on music perception and training of CI users understandably emphasizes perception in comparison to “normal” hearing; pitch and intervals are and will remain central to evaluating implant technology and the basic science of pitch coding. However, as one considers intrinsic reward of pattern acquisition in music (Gold et al., 2019), one can argue that “normal” pitch or interval perception, *alone*, may be too narrow an endpoint for evaluating perceptual learning and neuroplasticity.

Limitations to this study need to be discussed. Consistent with principles of qualitative methodology, this study was not intended to provide objective “truths” confirmed though testable hypotheses. This sample size was small and represents very specific characteristics. For example, these participants grew up in families with SES sufficient to afford and encourage active and sustained music enrichment. Despite assiduous recruitment efforts, this sample does not represent all music-centric pediatric CI users, nor all forms of music making. Future hypothesis testing with a larger sample is required to evaluate these factors, and generalizability of findings to other subgroups within the larger population of CI recipients. This will require addressing the financial and feasibility challenges associated with testing a dispersed patient population and conducting long-term training.

Future studies that examine music training in relation to auditory assessment would be valuable. In the present study, the auditory characteristics of the CI users were described through self-report and were general in nature, as opposed to detailed audiological assessments. For this study, perceptual measures were not obtained because of geographic distance (expense of and time for testing a geographically dispersed sample) and challenges in obtaining confidential data from patient files. Specific to the current study, it should also be noted that current audiological data would not have reflected specific changes in hearing status (e.g., mapping, signal processing, residual hearing) over the span of childhood and adolescence. In addition, audiograms predict only partially sound access and functional use of auditory input, and many centers do not routinely assess musically relevant stimuli.

We chose to focus on the music learning process as opposed to comparing device brands and signal processing. The CI users themselves did not emphasize brand-specific features of their own devices, and prior research has not documented clear advantage of specific brands for music; all CI manufacturers tout star users (Looi et al., 2012; Gfeller and Driscoll, 2020).

In summary, this study has examined the perceptual learning process of music-centric pediatric CI users who more nearly resemble experiences of trained musicians, as opposed to short-term music training of CI users (Gfeller, 2016). While the intensity and extensiveness of their training have yielded benefits in perceptual learning, social affiliation, and quality of life, researchers and clinicians are likely to think of the budgetary and feasibility challenges associated with providing training protocols of similar intensity and extensiveness (Gfeller, 2016; Torppa and Huotilainen, 2019). Furthermore, the sort of varied experiences described by these participants present challenges regarding confounding variables and examining causal relations. These are genuine concerns. Clean and tidy protocols have obvious advantages, but ecological validity is also a worthy goal.

Extended, intensive music engagement is a complex, and multifaceted experience; thus, psychosocial factors *are* likely to affect perceptual learning, and should be integrated into research protocols to increase motivation and reduce attrition. The most theoretically ideal training stimuli will not benefit a listener if they are not motivated to attend or persist. Accounting for influential psychosocial variables as part of analyses may also help account for variance and sharpen interpretation of results.

Finally, this study embraced patient-centered research methods in the hope of addressing more directly the concerns and needs of CI users who live each day with a valuable but imperfect hearing device. Even as researchers work in the long-term toward groundbreaking advances in signal processing and restoring damaged auditory structures, many CI users hope for practical strategies and (re)habilitative approaches in the near term, to experience more fully the joys of music in their daily life. The participants in this study, who have worked assiduously and with passion to (re)learn to hear music, offer insights that may open new paths for other CI users and for their healthcare providers.

## Data availability statement

The raw data supporting the conclusions of this article will be made available by the authors, without undue reservation.

## Ethics statement

The studies involving humans were approved by The University of Iowa Humans Subjects Research Committee. The studies were conducted in accordance with the local legislation and institutional requirements. The Ethics Committee/Institutional Review Board waived the requirement of written informed consent for participation from the participants or the participants’ legal guardians/next of kin because the study was deemed as exempt and low risk by the committee. That form is attached as Supplementary Information. Written informed consent was not obtained from the individual(s) for the publication of any potentially identifiable images or data included in this article because the project was approved by the IRB as exempt status and approval was implied by response to the questionnaire.

## Author contributions

KG: Conceptualization, Formal analysis, Funding acquisition, Investigation, Methodology, Project administration, Resources, Validation, Writing – original draft, Writing – review and editing. RM: Conceptualization, Investigation, Methodology, Writing – review and editing.

## Footnotes

1 Prestige musicianship for purposes of this paper, refers to advanced technical facility, auditory sensitivity and musicality required to play complex repertoire and to collaborate effectively (e.g., in tune, shared nuance of phrasing, interpretation) with other musicians.

2 Several of the participants implanted during adolescence were born in the 1990s. Candidacy for pediatric implantation has evolved since the 1990s. Consequently, using current candidacy guidelines, four of these participants [CI 2, 5, 6, P 2] may have been candidates for CIs at a much earlier age.

3 Testing of CI 2 in the first author’s lab confirmed pitch perception significantly below norms for typically hearing persons.

## Supplementary material

Supplementary Appendix A Questionnaire for music-centric pediatric CI users.

Supplementary Appendix B Questionnaire for parents of music-centric pediatric CI users.
