# Supplementary material for: Psychosocial and auditory factors that influence successful music-based auditory training in pediatric cochlear implant recipients
Source: Front Hum Neurosci. 2023 Dec 21;17:1308712. doi: 10.3389/fnhum.2023.1308712 (PMC10764544; doi:10.3389/fnhum.2023.1308712)
Supplement: Supplementary Appendix A — Questionnaire for music-centric pediatric CI users. [file Data_Sheet_1.pdf]

ID#CI__

Questionnaire for CI Users Implanted Before Age 18, and Actively Involved in Music While Growing Up

Before you fill out this questionnaire, we have two questions.

1. Did you get your first CI before the age of 18? Yes No

2. Were you actively involved in making music for at least 3 years while you were in junior high and/or high school? [This includes making music with friends (jamming), taking music lessons/classes, participating in ensembles] Yes No

If you did not answer yes to both questions, do not fill out the questionnaire. Please return the unanswered questionnaire to kay-gfeller@uiowa.edu. Thank you for considering participation in this study.

****

If you answered yes to both questions, continue with this form.

Why have we invited you to participate in this study?

- You were implanted with a CI before age 18.
- You have been actively involved in making and listening to music while growing up.
- Your musical involvement exceeded that of many CI users.
- We believe your experiences and ideas could help us understand what helps or hinders music enjoyment and involvement by CI users.
- Your experiences might help us to develop better programs for other CI users.

As you answer the questions below, keep these suggestions in mind:

- Share your experiences with music while you were growing up (through age 18).
- Please include positive, negative, or neutral experiences.
- **There are no right or wrong answers. We are interested in YOUR experiences as they happened.**
- You may have more you want to share on some questions. That is fine.

- You are free to skip any questions you prefer not to answer.
- **TO PROTECT YOUR PRIVACY AND THAT OF OTHERS, DO NOT INCLUDE THE NAMES OF ANY INDIVIDUALS IN YOUR RESPONSES. REFER ONLY TO TITLES (FOR EXAMPLE, MY BAND DIRECTOR, MY PARENT, MY AUDIOLOGIST, ETC.)**

HOW TO COMPLETE THIS QUESTIONNAIRE?

1. It may help to read all the questions first to decide which questions are most relevant to your life experiences.
2. Make an electronic copy of this document.
3. **Fill in the answer to the questions and save the document as MusicCI#3.doc.rev**
4. Email your questionnaire responses t**o** kay-gfeller@uiowa.edu

What will happen after I submit my questionnaire?

- You will receive a check for $25.00 as compensation for your effort.
- You may receive a follow-up email from Kate Gfeller. She may ask for clarification if she is not sure what one or more of your answers means. She wants to be sure that she understands correctly what you have shared.
- After that contact, your name and email address will be removed from the database to protect your privacy.

Thanks so much for helping us to understand the musical experiences of CI users.

Questionnaire

Please answer the following questions:

Note: Answer N/A for anything that does not apply to you.

Q1. Briefly describe you hearing loss and music experiences.

(a) Approximately how old were you when your hearing loss was diagnosed?

(b) Briefly describe your hearing loss (bilateral? progressive? Residual hearing on either side? etc.)

(c) Did you use a hearing aid before age 18? **Yes** **No**

If yes, how old were you? [for example, 2-8, 4-18] ____

(d) How old were you when you received your first implant? ______

2nd implant? _____

Type of implant(s)? ________________________________

(e). Do you use a hearing aid along with your CI? **Yes** **No**

(f) What instruments (voice) have you played, and for how long?

(g) Mark an X next to any of the following music activities you participated in. For each one that you check, describe how old you were while participating (e.g., 10-12; 8-10, etc.)

Music lesson? ______ ages ___________

Band or orchestra ______ ages ___________

Making music with friends (jamming, garage band, etc.)? _____ ages ________

Composing music (including sampling, synthesizer, etc.)? ______ ages _________

Dancing lessons or groups _______ ages __________

Other: ____ ages _____

Q 2. Describe what <u>informal</u> music experiences were like for you while growing up (no teachers, just for enjoyment).

- Some examples might include children's songs at bedtime, playing 'musical' toys, listening to music on your own, dancing/moving to music, going to concerts, jamming/playing music with friends, teaching yourself to play guitar, watching music videos, sharing play lists, using music apps (e.g., Spotify) to name a few.

- Indicate if your informal involvement with music changed at different stages in your childhood, (for example, during high school) or if you changed your hearing device (for example, hearing aid vs. CI).

Q3. In what ways, if any, did your friends [do not use names] influence your music involvement or preferences?

- For example, did your friends' musical habits or taste influence what you liked to play or listen to?

Q4. Describe any participation in formal music instruction (lessons, classes, ensembles) while growing up.

- Some examples would be music classes in school, music lessons, playing in ensembles or singing in choir in school, taking dance lessons, etc.

- Did your music participation change over time? Examples might include joining choir but quitting because it did not work out or starting one instrument but switching to another.

- If you were NOT involved in any formal music experiences, were there specific reasons?

    - Some examples might include that your school did not offer music classes, your family did not have money for lessons, or your Individual Education Plan had you attend speech therapy during music class.

Q5. If you <u>participated in</u> music classes or lessons, were there particular circumstances that either <u>helped</u> OR <u>hindered</u> your experiences in music?

- Were there situations or people [do not use names, only basic titles, such as music teacher, band director, audiologist, etc.] that helped you feel successful and encouraged you to stay in music?

- Did you figure out helpful strategies that helped you make music? (This might include using a visual tuning app to tune your instrument, tracking the percussion part to keep the tempo, etc.)

- Were there any situations or people [do not use names] that frustrated you, or made you want to quit music?

Q6. In what ways, if any, did your family's attitudes or interests (parents, siblings, grandparents) affect your musical experiences, instruction, and/or interests as you were growing up?

- Examples might be siblings or parents [do not use names] who played instruments, parents taking you to concerts, playing music in the household.

Q7. In what ways, if any, did you use technology (specific CI settings and strategies, apps, closed captioning, tuning instruments, assistive devices etc.) as part of music making or listening? Please describe specific apps, devices, or settings that you used, and how they helped.

Q.8 Were there any interactions, experiences, or circumstances that have been particularly frustrating, or that you feel have limited your satisfaction in playing or listening to music?

- What, if anything, helped you to persevere and move beyond these frustrations and limitations?

Q9. Describe beliefs/attitudes that you or others had that motivated you to stay involved in music making or listening enjoyment.

Q10. Based on your experiences, what advice, if any, would you give a young person growing up with a CI about enjoying music and cochlear implants?

Q11. What else, if anything, do you want us to know/ understand about music experiences and growing up with a CI?

Q 12. What does making and listening to music ‘do’ for you? Another way of asking that question might be “what do you ‘get’ from making and listening to music? Or how does making and listening to music affect you?

- This might be a bit hard to put into words. . . . there is no right or wrong answer. This is a very individual thing.
