# Supplementary material for: Psychosocial and auditory factors that influence successful music-based auditory training in pediatric cochlear implant recipients
Source: Front Hum Neurosci. 2023 Dec 21;17:1308712. doi: 10.3389/fnhum.2023.1308712 (PMC10764544; doi:10.3389/fnhum.2023.1308712)
Supplement: Supplementary Appendix B — Questionnaire for parents of music-centric pediatric CI users. [file Data_Sheet_2.pdf]

ID#P__

Questionnaire for Parents of Cochlear Implant (CI) Users Implanted Before Age 18, and

Actively Involved in Music While Growing Up

Before you fill out this questionnaire, answer these two questions:

1. Did your child receive their first CI before the age of eighteen? **Yes** **No**

2. Are/Were they actively involved in making music for at least 3 years while they were in junior high and/or high school? [This includes making music with friends (jamming), taking music lessons/classes, participating in ensembles] **Yes** **No**

If you did not answer yes to both questions, do not fill out the questionnaire. Please return the unanswered questionnaire to kay-gfeller@uiowa.edu. Thank you for considering participation in this study.

****

If you answered yes to both questions, continue with this form.

Why have we invited you to participate in this study?

- Your child was implanted with a CI before age 18.
- Your child has been actively involved in making and listening to music while growing up.
- We believe your experiences could help us understand what helps or hinders music enjoyment and involvement by CI users.
- Your experiences might help us to develop better programs for other CI users.

As you answer the questions below, keep these suggestions in mind:

- Share your child's experiences with music while your child is/was growing up (through age 18). Feel free [as suits your situation] to discuss with your child on these questions as you prepare your responses.

- Please include positive, negative, or neutral experiences.
- **There are no right or wrong answers. We are interested in YOUR child's experiences as they happened.**
- You may want to write more detail on some questions. That is fine.
- You are free to skip any questions you prefer not to answer.
- **TO PROTECT YOUR PRIVACY AND THAT OF OTHERS, DO NOT INCLUDE THE NAMES OF ANY INDIVIDUALS IN YOUR RESPONSES. REFER ONLY TO TITLES (FOR EXAMPLE, MY BAND DIRECTOR, MY PARENT, MY AUDIOLOGIST, ETC.)**

HOW TO COMPLETE THIS QUESTIONNAIRE?

1. It may help to read all the questions first to decide which questions are most relevant to your life experiences.
2. Make an electronic copy of this document.
3. **Fill in the answer to the questions and save the document as MusicP_ID4.doc.rev**

4. Email your questionnaire responses t**o** kay-gfeller@uiowa.edu

What will happen after I submit my questionnaire?

- You will receive a check for $25.00 as compensation for your effort.
- You may receive a follow-up email from Kate Gfeller. She may ask for clarification if she is not sure what one or more of your answers means. She wants to be sure that she understands correctly what you have written.
- After that contact, your name and email address will be removed from the database to protect your privacy.

Thanks so much for helping us to understand the musical experiences of CI users.

****

Questionnaire

Please answer the following questions [Note: Answer N/A for anything that does not apply to you.]

Q1. Briefly describe your child's hearing loss and music experiences.

(1a) How old was your child when their hearing loss was diagnosed?

(1b) Briefly describe your child's hearing loss (bilateral? progressive? Residual hearing on either side? etc.)

(1c) Did your child use a hearing aid before age 18? **Yes** **No**

If yes, how old are/were they? [for example, 2-8, 4-18] ____

(1d) At what age did your child receive the first implant? ______

2nd implant? _____

Type of implant(s)? ________________________________

(1e). Does your child use a hearing aid along with their CI? **Yes** **No**

(1f) What instruments (voice) did/does your child played, and for how long?

(1g) Mark an X next to any of the following music activities your child participated in. For each one that you check, describe how old your child was while participating (e.g., 10-12; 8-10, etc.)

- Music lesson? ______ ages ___________
- Band or orchestra ______ ages ____________
- Making music with friends (jamming, garage band, etc.)? _____ ages ________
- Composing music (including sampling, synthesizer, etc.)? ______ ages _________
- Dancing lessons or groups _______ ages __________
- Other: ____ ages _____

Q 2. Describe what <u>informal</u> music experiences (no teachers, just for enjoyment) were like for your child while growing up.

- Some examples might include children's songs at bedtime, playing 'musical' toys, listening to music on your own, dancing/moving to music, going to concerts, jamming/playing music with friends, teaching yourself to play guitar, watching music videos, sharing play lists, using music apps (e.g., Spotify) to name a few.

- Indicate if their informal involvement with music changed at different stages of childhood, (for example, during high school) or if they changed hearing devices (for example, hearing aid vs. CI).

Q3. In what ways, if any, did your child's friends [**do not use names**] influence their music involvement or preferences?

- For example, did their friends' musical habits or taste influence what they liked to play or listen to?

Q4. Describe any participation by your child in formal music instruction (lessons, classes, ensembles) while growing up.

- Some examples would be music classes in school, music lessons, playing in ensembles or singing in choir in school, taking dance lessons, etc.

- Did their music participation change over time? Examples might include joining choir but quitting because it did not work out or starting one instrument but switching to another.

- If they were NOT involved in any formal music experiences, were there specific reasons?

    - Some examples might include that your school did not offer music classes, your family did not have money for lessons, or your child's Individual Education Plan had speech therapy during music class.

Q5. If your child was/has been involved in music classes or lessons, what circumstances either helped OR hindered experiences in music?

- Were/Are there situations or people [**do not use names,** only basic titles, such as music teacher, band director, audiologist, etc.] that helped your child feel successful and encouraged to stay in music?

- Did your child figure out strategies that helped them make music? (This might include using a visual tuning app to tune your instrument, tracking the percussion part to keep the tempo, etc.)
- Were there any situations or people [**do not use names**] that frustrated your child, or made them want to quit music?

Q6. Were there any family attitudes or interests (parents, siblings, grandparents) that you believe affected your child's musical experiences, instruction, and/or interests while growing up?

- Examples might be siblings or parents [**do not use names**] who played instruments, family outings to concerts, playing music in the household.

Q7. In what ways, if any, did your child use technology (specific CI settings and strategies, apps, closed captioning, tuning instruments, assistive devices etc.) as part of music making or listening? Please describe specific apps, devices, or settings, and how they helped.

Q.8 Were there any interactions, experiences, or circumstances you are aware of that were particularly frustrating, or that you feel have limited your child's satisfaction in playing or listening to music?

- What, if anything, helped your child to persevere and move beyond these frustrations and limitations?

Q9. Describe beliefs/attitudes that your child or others had that motivated your child to stay involved in music making or listening enjoyment.

Q10. Based on your experiences, what advice, if any, would you give a parent of a young person growing up with a CI or a child with a CI about enjoying music and cochlear implants?

Q11. What else, if anything, do you want us to know/ understand about music experiences and growing up with a CI?
