# Supplementary material for: Psychosocial and auditory factors that influence successful music-based auditory training in pediatric cochlear implant recipients
Source: Front Hum Neurosci. 2023 Dec 21;17:1308712. doi: 10.3389/fnhum.2023.1308712 (PMC10764544; doi:10.3389/fnhum.2023.1308712)
Supplement: Supplementary file 3 [file Data_Sheet_3.pdf]

FOR IRB USE ONLY
APPROVED BY: IRB-01
IRB ID #: 202302644
APPROVAL DATE: 04/19/23

Project Title: **Music Engagement of Cochlear Implant Recipients Who Grew Up with Cochlear Implants**

Principal Investigator: Kate Gfeller

We invite you to participate in a research study being conducted by investigators from The University of Iowa. The purpose of the study is to understand more fully the active music experiences of young adults who grew up using cochlear implants (CI), and whose perception of and involvement in music has exceeded typical music involvement of CI users reported in the research literature. We are interested in technological, psychological, environmental, familial, educational, and societal factors that have supported or functioned as barriers to active involvement in music.

If you agree to participate, we would like you to fill out a questionnaire that asks questions about you or your child's experiences with music while growing up, and while using a cochlear implant. You are free to skip any questions that you prefer not to answer. It will take approximately one hour to fill out the questionnaire.

As a thank you for your time and effort, you will be compensated with $25.00 upon submitting the questionnaire to the researcher.

In order to protect your confidentiality, we will not collect your name or any directly identifying information about you. We also will ask that you not include the names of other people in your responses.

Taking part in this research study is completely voluntary. If you do not wish to participate in this study, return the survey without answering any of the questions. There will be no risk to your typical care or access to services.

If you have questions about the rights of research subjects, please contact the Human Subjects Office, 105 Hardin Library for the Health Sciences, 600 Newton Rd, The University of Iowa, Iowa City, IA 52242-1098, (319) 335-6564, or e-mail irb@uiowa.edu.

We encourage you to ask questions. If you have any questions about the research study itself, please contact: Kate Gfeller, Ph.D., The University of Iowa, phone: 319-321-0342.

Thank you very much for your consideration of this research study.
